# Decisions about the use of psychotropic medication during pregnancy: a qualitative study

**DOI:** 10.1136/bmjopen-2015-010130

**Published:** 2016-01-27

**Authors:** Fiona Stevenson, Sarah Hamilton, Vanessa Pinfold, Charlotte Walker, Ceri R J Dare, Harminder Kaur, Ruth Lambley, Paulina Szymczynska, Vicky Nicolls, Irene Petersen

**Affiliations:** 1 Department of Primary Care and Population Health, UCL, London, UK; 2 McPin Foundation, London, UK; 3 Unit for Social and Community Psychiatry, Queen Mary University of London, London, UK; 4 Mental Health Foundation, London, UK; 5 Department of Clinical Epidemiology, Aarhus, Denmark

**Keywords:** MENTAL HEALTH, DECISION MAKING, MEDICINE TAKING, QUALITATIVE RESEARCH, HEALTH EXPERIENCES

## Abstract

**Objective:**

To understand the perspectives of women with severe mental illness concerning the use of psychotropic medicines while pregnant.

**Design:**

Interviews conducted by female peer researchers with personal experience of making or considering decisions about using psychotropic medicines in pregnancy, supported by professional researchers.

**Participants:**

12 women who had had a baby in the past 5 years and had taken antipsychotics or mood-stabilisers for severe mental illness within the 12-month period immediately prior to that pregnancy. Recruitment to the study was via peer networks and the women interviewed came from different regions of England.

**Setting:**

Interviews were arranged in places where women felt comfortable and that accommodated their childcare needs including their home, local library and the research office.

**Results:**

The views expressed demonstrated complex attempts to engage with decision-making about the use of psychotropic medicines in pregnancy. In nearly all cases, the women expressed the view that healthcare professionals had access to limited information leaving women to rely on experiential and common sense evidence when making decisions about medicine taking during pregnancy.

**Conclusions:**

The findings complement existing work using electronic health records by providing explanations for the discontinuation of psychotropic medicines in pregnancy. Further work is necessary to understand health professionals’ perspectives on the provision of services and care to women with severe mental illness during pregnancy.

**Strengths and limitations of this study**

- Women with personal experience of using antipsychotic medications and making or considering decisions about medicines and pregnancy, supported by professional researchers, developed the research questions, designed the study and conducted the interviews.
- Women with personal experiences of decisions about the use of antipsychotic medications were involved in the analysis; grounding the findings in patient experience.
- The findings are based on what the women recalled about their decision-making at that time.
- Further work is needed to consider women's accounts while pregnant, the perspectives of healthcare professionals, fathers and other family members.

## Introduction

Many women with severe mental illness treated with psychotropic medication become pregnant.^1–3^ However, no psychotropic medication has UK marketing authorisation specifically for pregnant women or for use when breast feeding.^4^ ^5^ This leaves women and their healthcare professionals in a treatment dilemma in relation to the use or otherwise of psychotropic medicine in pregnancy, with the need to balance the health of the women with that of the unborn child without recourse to an established evidence base.^6–9^ Advice on treatment varies across countries and in some instances standard psychiatric advice is that women maintain pharmacological treatment across the perinatal period.^10^ The 2014 National Institute for Health and Care Excellence (NICE) guidelines for antenatal and postnatal mental health provide some guidelines for specific treatments, such as lithium and valproate, but the evidence base for other treatments is limited.^4^ The higher threshold for starting any treatment in pregnancy is, however, noted.^4^

Analysis of data from established electronic health records such as The Health Improvement Network (THIN) and the Clinical Practice Research Datalink (CPRD) primary care databases, as well as US insurance claims databases, shows pregnancy to be a key determinant for stopping most psychotropic medications.^11–17^ Petersen *et al*^14^ showed that this was the case for antidepressants; with the decline in prescribing the most profound in the first 6 weeks of pregnancy. A similar pattern was observed for antiepileptic drugs, with pregnancy a determinant for discontinuation, particularly for women with bipolar disorder or depression.^12^ Focusing on antipsychotics, <40% of women who received atypical antipsychotics prior to pregnancy were still in receipt of them at the start of their third trimester. For typical antipsychotics, the figure was down to 19% by the start of the third trimester,^11^ and for lithium the figure was 29%.^18^ It has, however, been argued that although reductions in prescribing of psychotropic medicines in pregnancy might be explained by concerns about adverse effects of the drugs, this needs to be balanced against the potential harm of inadequate treatment during pregnancy.^9^ ^12^ ^14^ ^19–21^ From the perspective of women, analysis of a Norwegian questionnaire study involving pregnant women, or those with a child under the age of five, found that the harmful effect of all drugs in pregnancy was overestimated, with the risks of antidepressants perceived to be on a par with alcohol, smoking and thalidomide.^22^ Similar findings have been reported in studies based in Europe, North America and Australia.^23^

Overall, little is known about the real-world decision-making processes navigated by women and their healthcare providers around whether to initiate and/or continue psychopharmacology during pregnancy and about how women weigh and consider information, personal beliefs, life experiences and perceptions of their mental health needs and treatment preferences to make decisions about their use of psychiatric medication.^24^ The recently updated NICE guidelines^4^ state that the prescriber should take into account individual needs and preferences and that women should have the opportunity to make informed decisions about their care and treatment, in partnership with healthcare professionals. Discussions should involve the potential benefits of psychological interventions and psychotropic medication, the possible consequences of no treatment, the possible harms associated with treatment and what might happen if treatment is changed or stopped, particularly if psychotropic medication is stopped abruptly.^4^ Moreover, the complexity, and associated uncertainty, of making decisions about medicine taking during pregnancy for women with mental health problems is explicitly acknowledged in relation to judging where changes in mental health state and functioning (such as appetite) may represent normal pregnancy changes or alternatively may be a symptom of a mental health problem.^4^ All of this fits with the need to work with women rather than merely applying a model of risk management, with the importance of such an approach also noted from the perspective of service users.^25^

The research presented here provides women's accounts of decisions concerning the use of psychotropic medicines while pregnant, complementing and extending findings from studies that used electronic health records and questionnaire surveys. It is vital to understand the perspectives of women involved in such decisions as they are the ones that live with the consequences of decisions about medicine taking in pregnancy both during and after pregnancy.

## Methods

Four women with personal experience of using antipsychotic medications and making or considering decisions about medicines and pregnancy were recruited as peer researchers. They were supported by professional researchers to develop research questions and design the study. Research methods training was provided to the four peer researchers. Twelve women who had had a baby in the past 5 years and had taken antipsychotics or mood-stabilisers for severe mental illness within the 12-month period immediately prior to that pregnancy were interviewed. Interviews were conducted in pairs; one person led the interview while the other took charge of the recording equipment and other practical issues. Interviews generally lasted for about 50 min, with the shortest being 24 min and the longest 2 h 20 min. Recruitment to the study was via peer networks and the women interviewed came from different regions of England. The analysis and write-up stages were led by professional researchers with peer involvement.

The interviews explored women's accounts of pregnancy and decisions about medicine taking. Women's mental health at the time of the pregnancy, the formal and informal support received, use of medicines at that time, their history of medicine taking and views and beliefs about medicines were also explored. Women were asked specifically about any changes made to medicines when they were pregnant, how those decisions were reached and if there was anything that might have made decisions easier at this time. The topics covered were developed by the peer researchers, with guidance from the professional researchers. Interviewees raised the issue of decisions about infant feeding in early interviews and this was subsequently incorporated into the topic guide.

Interviews were arranged in places where women felt comfortable being interviewed and that accommodated their childcare needs. They were conducted in women's own homes, a local library, rented office space and the charity's research office. All interviews started by asking women to reflect on what was going on in their lives when they first found out they were pregnant, with our specific areas of interest introduced as prompts as the interview developed. This was a pilot study with sufficient resources to conduct 12 interviews. All interviews were fully transcribed and subject to thematic analysis. Each of the peer and professional researchers examined three transcripts in detail and developed their own coding framework. These were examined in a half-day data clinic in which all the team members (professional and peer researchers) presented their individual analyses. A joint coding framework was then agreed on and applied to the data. For the purposes of this paper, an additional analysis focusing on women's accounts of involvement in decision-making about medicine taking in pregnancy, specifically in relation to accounts of the evidence, formal and informal, used to support decisions, was led by a professional researcher, with comments from the rest of the team.

## Findings

Key characteristics of the sample are provided in table 1. The accounts provided by the women in the study are considered in relation to information-seeking behaviour, the use of experiential evidence and the effects of access to services on women's medicine taking.

**Table 1:** Key characteristics of women interviewed (n=11)*

Characteristics	Count	
Age	Range 28–46 years Mean 37.45	
Ethnicity	White British	8
	White other	2
	Arab	1
Place of residence	London	7
	North West	1
	Yorkshire and North East	2
	Eastern	2
Number of children	Range 0–3 Mean 1.6	
Diagnosis	Schizophrenia/schizoaffective	3
	Bipolar	8
Current service support for mental health	General practitioner	3
	Secondary care services	6
	Data not supplied	2
Psychotropic medication use during pregnancy	Took no psychotropic medication	2
	Medication in trimester one	9
	Medication in trimester two	8†
	Medication in trimester three	7†
Planning of pregnancy	Planned	6
	Unplanned	5
	Unclear	1

*One participant declined to complete a participant profile form.

†In addition, one woman took antidepressants.

### Information seeking

As can be seen in table 1, six women reported that their pregnancies were planned, five as unplanned, and in one interview it was unclear. Of those who reported that their pregnancies were planned, five of the six reported discussing this with a medical practitioner prior to the pregnancy. One woman reported having discussed the possibility of getting pregnant with her doctor at some period in the past, but her pregnancy was unplanned. Another woman reported having done a lot of research but did not discuss the role of the medical profession as part of that research; a possible explanation for this was that she was not using any services at the time. In summary, the women in our sample reported actively seeking advice prior to a planned pregnancy and in general were aware of their mental health needs and keen to be involved with decisions that affected their health.

It was, however, generally agreed that there was a lack of evidence available in relation to the risks and benefits of taking psychotropic medicines in pregnancy. Consequently, even where women reported feeling supported, decisions were described as taking a ‘punt’ or ‘gamble’.

You have to make the decision based on no information really (P05)

Yet for women making decisions, the stakes in relation to their own health and that of their unborn child are very high. Although some women did discuss the role of their partner and wider friends and family in providing support, only two presented and discussed their decisions in such a way as to indicate joint decisions.

Criticism was directed at doctors who provided definitive answers, not just because of the dearth of evidence but particularly where this did not allow for discussion of the basis for advice and associated uncertainty. This was presented as particularly problematic as it assumes women and medical professionals judge risks and benefits and the inevitable trade-offs in the same way.

I think clinicians can sometimes feel the need to give what sounds like a definitive answer, even if there isn't one, and actually it would be better to say there isn't enough research yet (P08)

In contrast, failure to provide a strong direction could mean people felt they had to ‘resort’ to seeking guidance from the internet.

If she'd have said I'd rather you didn't take it then I would have stopped it, but she was just very wishy washy…So I then went and you know had a look on the internet to see what was going on (P07)

Most of the women in our study indicated that they had used the internet to search for information about the use and effects of psychotropic drugs in pregnancy. The internet was used to search for research papers, information about the effects on child development of mothers taking psychotropic medicines in pregnancy, and information about different formulations of psychotropic medicines. Some women presented accounts about use of the internet in general suggesting that they probably had used it when considering the use of psychotropic medicines in pregnancy based on the fact they that had used it for information about medication in the past. This lack of specific detail may be indicative of the normalisation of internet use for many in society. Information from the internet was, however, criticised for being too vague and for being inconsistent. The contrast between information presented in National Health Service (NHS) guidelines and what was generally available online was noted; in particular that guidelines present information as cut and dried, while the internet allows access to a range of views, opinions and experiences. Some women were appreciative of the fact that the internet gave them access to a range of information and evidence, despite the fact that it may be contradictory.

With the NHS, there are the guidelines and it's very cut and dry isn't it? And they will say to you, well you can take it but the risk has got to outweigh the benefits, blah di blah blah, and that's all you ever hear. So there's lots of these sites where someone will post a question and then an American psychiatrist will answer it and they'll sort of give more their opinion or you know things that they've seen…And some people said…my baby's fine, my pregnancy was fine, no problems whatsoever. And then others are saying things like, oh my baby's behind or he's got autism, my baby was born with disabilities (P07)

Print media was also discussed as a source of information. One woman indicated that negative reports reinforced her decision not to take medicines, while for another absence of reports was taken as indicative of the fact that a particular medicine cannot be too dangerous. Finally, in another example, the arguments presented in a book were expressed as helpful by one woman in enabling her to live with her decision to take medicines throughout her pregnancy.

### Experiential evidence

Experiential evidence may be central to women's decisions. This evidence may take the form of women's own experiences, or women may draw on the lived experiences of others through, for example, the internet.

The need to control symptoms, based on experiences when not taking medicines, was presented as the rationale for some women to stay on medicines while pregnant.

And how did you feel at that point about drugs in pregnancy, did you have any kind of preconceptions or…?

No, I mean having had the experience that I'd had and trying to do it without medication and finding that it didn't work, for me, then I sort of felt like it was important for me to stay on a stabilising dose of Prochlorperazine and that…that if that kept me stable, that was less of a risk to me and the baby and all that kind of thing. So I didn't know much about, there may be all sorts of medical things that I'm ignorant of, in terms of you know the effect on the foetus or whether or not it's best to be off them altogether. But I just felt for me, practically, I wasn't going to get through the pregnancy without having some kind of stabilising influence (P09)

Other women drew on previous experiences of pregnancy. Experiences were presented as supporting decisions to take and not to take medicines. In the most extreme case in our sample, medical guidelines were reported to have been used to override experiential evidence when a doctor reportedly would not allow a nurse to give a pregnant woman a routine depot injection despite the woman in question having received these injections throughout her previous pregnancy.

I was like no, because I have a child who is four years old and I know from my experience that I can't come off my medication, I know that because I've had one child, throughout my whole pregnancy I've been on my depot and I know how it works because my old doctor, I've been with him, so he's known me since I was like 14, I was on the same medication throughout the pregnancy, and I know not to come off it (P10)

She reported becoming so ill following the withdrawal of treatment that she had an abortion. She went on to describe how she subsequently moved into the area covered by her previous mental health team. Her treatment was then changed from depot injections to tablets and following this change she went on to have a baby. She presented her experience with her first child as evidence that she could continue taking medicine throughout pregnancy without it necessarily having an adverse effect.

Similarly, another woman reported that by the time of her second pregnancy she had come to terms with the necessity of taking medicines throughout pregnancy in order to ‘stay well’. The ‘moral challenge’ potentially presented by the use of medicines in pregnancy was directly countered by the appeal to medicines allowing her to stay well and ‘be a good mum’.

Did how you felt about your meds change when you found you were pregnant or…?

No, because I think this time round and the whole sort of process that I'd gone through to that point…my views of the illness had changed by that time and my thinking, which is the same as now really, is that it's much more of a chemical problem…that is something beyond what I can kind of mentally sort of overcome myself, that there's actually some sort of fundamental chemical problem that nothing other than the meds seems to be able to sort out. I've had all kinds of talking therapies and so on and so on and really haven't made a difference. But…And I find it hard to sort of believe that taking a couple of little tablets makes such a huge difference to the way that I function, but…My thinking now is I clearly need them and I want to just stay well and be a good mum (P02)

In contrast, a different woman who had previously had a stillborn baby presented the protection of her unborn baby from the potential effects of medication as key.

One of the main decisions what influenced me not going back on medication was because I wasn't going to take anything that risked D in the…in my womb. I'd already lost one child…I wasn't going to risk losing another. So for me, I didn't trust medi…in medication, and I had to trust in myself (P04)

She also described a side effect of her antipsychotic medicines to be a muting of her emotional reactions, expressing her desire to know what it felt like to hold her child and emotionally bond as an additional reason to avoid taking medicines while pregnant and immediately after.

Finally, in the absence of other information, formal, informal or experiential, women reported making decisions based on the age and dose of medicines and the common sense idea that a lower dose of an established medicine was likely to be a lower risk than a newer medicine about which little was known.

### Impacts of access to services

Discussion of decisions about psychotropic medicine in pregnancy cannot be divorced from the context and circumstances in which services are provided. For example, one woman described working with her psychiatrist to stop taking medicines so she could become pregnant, but when she succeeded in stopping taking the medicine she was discharged from the service with the result that she had no support from her psychiatrist through her pregnancy. In another case, the account was of abandonment and gradual deterioration, with treatment not offered when health deteriorated.

Now in the first trimester, I felt it was important to safeguard, to prevent the baby from having any of the side effects, damage from the medication, while it's forming. But by the third trimester or the second trimester even, my CPN [community psychiatric nurse] had recognised I was showing, displaying signs of psychosis…nothing was done (P12)

A particular problem related to the time it took for referrals; leading to reports of medicine being either reduced or stopped without support or of women taking medicine by default through the first trimester of pregnancy.

Reports of relationships with medical professionals were mixed. Some women described collaborative working, while others reported being made to feel guilty for wanting to continue or to stop medicines. As an example, one woman described the focus placed by health professionals on the risk of relapse and attending classes to support parenting.

I found that because I were choosing to stay off medication, I were constantly being told, you're 50 percent chance likely to relapse. And I found that because I didn't go on meds, they made me do God knows how many different positive parenting classes to prove that I could be a mum (P04)

The organisation of healthcare can also have profound consequences for pregnant women with long-term mental health problems. Thus, experiences were related of being on maternity wards on which there was no understanding of mental health issues and of being on a psychiatric ward where pregnancy was not supported. These experiences obviously have consequences for the provision of care.

## Discussion

Medicine taking in pregnancy is perceived as risky,^22^ ^23^ yet little is known about the real-world decision-making processes navigated by women and their healthcare providers around whether to initiate and/or continue psychotropic medicines during pregnancy and about how women weigh and consider information, personal beliefs, life experiences and perceptions of their mental health needs and treatment preferences to make decisions about their use of psychiatric medication.^24^ This paper presents women's accounts of decisions about medicine taking and the complexity of balancing the risks to their own mental health against possible risks to their unborn child. In nearly all cases, the women expressed the view that healthcare professionals struggled to find definitive evidence on which to base their recommendations, leading in some cases to interactional difficulties in the healthcare professional—patient relationship at a crucial time in their lives. Women were left to draw on experiential and common sense evidence and then to ‘take a punt’ on decisions about getting pregnant and the use of psychotropic medicines in pregnancy. The overriding discourse was about the complexity of women's situations in relation to their health and requirement of psychotropic medication to stabilise their symptoms, potential consequences for their unborn child, and the level of support, or otherwise, both formally and informally available.

### Strengths and weakness

This paper outlines the real-world decision-making processes navigated by women making decisions about the use of psychotropic medication in pregnancy, which has been identified as a gap in the literature.^24^ It provides accounts of the extent to which women felt able to make an informed choice. It does, however, draw on a small number of participants (12 in total) and the interviews provide a retrospective account of their decisions in pregnancy; therefore, the accounts provided may have been affected by subsequent events, in particular the health of their baby and themselves. Moreover, we have no information from women who, having considered the potential consequences for their health of pregnancy, have decided not to have children.

### Future work

Complexity and uncertainty were recurrent themes in women's accounts of their interactions with healthcare professionals, suggesting the need for further work to consider the perspectives of a range of healthcare professionals and how they feel about advising women about the use of psychotropic medicines in pregnancy. Further work is also needed to consider women's accounts while pregnant and also the perspectives of fathers and other family members. This would complement work using prescribing databases to track changes in prescribing patterns related to pregnancy. Our findings also highlight the need for more detailed research on the information available on the internet in relation to the use of psychotropic drugs and use of the internet for this purpose.

## Conclusions

The findings reported here provide women's perspectives on real-world decisions made about the use of psychotropic drugs in pregnancy. They provide an understanding of the complexity of decision-making in such circumstances and challenges inherent in making informed decisions, given the lack of research-based evidence on the effects of taking psychotropic drugs in pregnancy. The findings complement existing work using prescribing databases by providing explanations for the discontinuation of psychotropic medicines in pregnancy and identify the need for further work in order to support health professionals in the provision of services and care to women with long-term mental health problems during pregnancy.
